# Comparison of triploid and diploid rainbow trout (*Oncorhynchus mykiss*) fine-scale movement, migration and catchability in lowland lakes of western Washington

**DOI:** 10.1186/s40462-023-00418-w

**Published:** 2023-09-15

**Authors:** Jessica E. Pease, James P. Losee, Stephen Caromile, Gabriel Madel, Michael Lucero, Anna Kagley, Michael G. Bertram, Jake M. Martin, Thomas P. Quinn, Daniel Palm, Gustav Hellström

**Affiliations:** 1 Washington Department of Fish and Wildlife, OlympiaWashington, WA USA; 2 Department of Wildlife, Fish and Environmental Studies, Swedish University of Agricultural Sciences, Umeå, Sweden; 3 NOAA Fisheries, Northwest Fisheries Science Center, Seattle, WA USA; 4 School of Aquatic and Fishery Sciences, University of Washington, Seattle, WA USA; 5 Department of Zoology, Stockholm University, Stockholm, Sweden; 6 School of Biological Sciences, Monash University, Melbourne, Australia

**Keywords:** Introgression, Creel, Angler catch rates, Telemetry, Stocking

## Abstract

Fisheries managers stock triploid (i.e., infertile, artificially produced) rainbow trout *Oncorhynchus mykiss* in North American lakes to support sport fisheries while minimizing the risk of genetic introgression between hatchery and wild trout. In Washington State, the Washington Department of Fish and Wildlife (WDFW) allocates approximately US $3 million annually to stock hatchery-origin rainbow trout in > 600 lakes, yet only about 10% of them are triploids. Many lakes in Washington State drain into waters that support wild anadromous steelhead *O. mykiss* that are listed as threatened under the U.S. Endangered Species Act. As a result, there is a strong interest in understanding the costs and benefits associated with stocking sterile, triploid rainbow trout as an alternative to traditional diploids. The objectives of this study were to compare triploid and diploid rainbow trout in terms of: (1) contribution to the sport fishery catch, (2) fine-scale movements within the study lakes, (3) rate of emigration from the lake, and (4) natural mortality. Our results demonstrated that triploid and diploid trout had similar day-night distribution patterns, but triploid trout exhibited a lower emigration rate from the lake and lower catch rates in some lakes. Overall, triploid rainbow trout represent a viable alternative to stocking of diploids, especially in lakes draining to rivers, because they are sterile, have comparable home ranges, and less often migrate.

## Introduction

Fisheries managers have stocked rainbow trout *Oncorhynchus mykiss* in rivers and lakes to support conservation and recreational objectives for over a century [34]. The native range of rainbow trout is restricted to western North America and eastern Russia, but rainbow trout currently inhabit much of the world and persist as self-sustaining populations outside the native range as a result of these stocking programs [8, 31]. However, there is also extensive stocking within their native range. For example, over 2 million rainbow trout are stocked annually in Washington State, USA [41].

Rainbow trout stocking has been linked to important conservation gains [1, 10], and significant economic benefits [17]. For example, in Washington State, rainbow trout stocking is responsible for over US$1.1 billion of revenue [11]. However, in many parts of the world, there has been growing concern that stocked rainbow trout pose potential risks to natural ecosystems through competition, predation, and spawning with native species [8, 23, 26]. In the United States, introgression between stocked rainbow trout and with natively threatened anadromous rainbow trout (steelhead) and coastal cutthroat trout, *O. clarkii clarkii*, is a major issue for maintaining genetic integrity and overall fitness [14, 30, 38, 43]. However, given funding limitations and public satisfaction with rainbow trout stocking programs, formal evaluation of the costs and benefits of these popular programs are lacking [4, 39].

One strategy that managers use to reduce hybridization between native and hatchery-origin fish is to stock sterile, triploid rainbow trout rather than traditional diploids, particularly in lakes draining into waters accessible to anadromous conspecifics (i.e., wild steelhead listed as *Threatened* under the Endangered Species Act in the Puget Sound region of Washington, and elsewhere). For instance, the state of Idaho adopted a policy in 2001 stocking only sterile, not diploid, rainbow trout in flowing waters. In Washington State, where most steelhead populations are listed as *Threatened*, the WDFW allocates approximately US$3 million annually to stock hatchery-origin rainbow trout in > 600 lakes but less than 10% of the fish stocked are triploids [11]. Increasing the use of triploid trout in popular trout fisheries may help conserve the genetic integrity of native populations but the effect on catch rates is unclear. For instance, Dillon et al., [9] found no significant differences in catch rate and fishery duration between the two trout ploidy strains in Idaho streams. On the other hand, Koenig et al. [21] and Koenig and Meyer [22] documented differences in survival across habitat conditions and higher catch rates of diploid than triploid trout in lake systems. Differences between triploid and diploid rainbow trout catchability are poorly understood and difficult to assess but could include different rates of survival, migration from the lake, and feeding, and in-lake movement patterns. To ensure conservation objectives while maintaining successful fisheries when switching from diploid to triploid rainbow trout, post-stocking mortality, migration rate, and recruitment to the fishery of triploids and diploids need to be compared.

Many tools have been developed to assess individual fish movements, growth, and survival, including a variety of tags, transmitters, and marking techniques [5, 27]. Acoustic telemetry has accelerated research on fish behavior as it can reveal patterns of fish behavior, habitat use, predation, and migration [5, 6, 19, 24]. For example, acoustic telemetry has revealed precise survival rates of stocked rainbow trout in rivers and lakes, interactions with natural populations, and diel movement patterns [16, 20, 40]. The uncertainty around the catchability and movement patterns of triploid trout in popular sport fisheries and the potential for these sterile fish as an alternative to traditional stocking of diploid trout objectives make acoustic telemetry a suitable assessment technique, especially if paired with studies on the catchability and movement patterns of triploid and diploid trout. Accordingly, the objectives of this study were to compare diploid and triploid rainbow trout with respect to their (1) contribution to lake sport fisheries, (2) fine-scale movements in the lake, (3) rate of migration from the lake, and (4) natural mortality. Movements patterns of stocked diploid and triploid trout revealed in this study will improve the ability of inland fisheries managers to maximize catch rates or rainbow trout while meeting management objectives associated with conservation.

## Methods

### Creel sampling

Goldendale, fall spawning strain, triploid (mixed sex, thermally heat shocked) and diploid rainbow trout were reared to similar size at Eels Springs Hatchery in Shelton, Washington on spring water. Equal numbers of triploid and diploid trout (36,372 of each) were stocked into 15 western Washington lakes (Table 1) to achieve a ratio of 50:50 triploid to diploid, targeting a total stocking density of 22.26 fish/hectare (Table 1). Triploid trout were marked for field identification by removing the adipose fin 6 months prior to stocking. Stocked trout fell within the “catchable” size with a stocking rate of 1.04 fish per kilogram ± 0.03 SD (mean ± SD; triploid = 1.05 ± 0.03 and diploid = 1.04 ± 0.03). All fish were stocked 1 week prior to the opening day of trout season (24 April 2021).

**Table 1:** Surface Hectare of studied lakes and stocking density of triploid and diploid trout in western Washington prior to opening day of trout fishing (April 24th) in 2021

Lake name	County	Size	Number of fish stocked			Stocking density
		Surface hectare	Triploids	Diploids	Total	Fish/Hectare
Clear Lake	Thurston	70	4760	4760	9520	136.0
Hicks Lake	Thurston	65	4400	4400	8800	135.9
Ward Lake	Thurston	27	1835	1835	3670	135.4
Crescent Lake	Pierce	19	1293	1293	2586	136.0
Ohop Lake	Pierce	96	6490	6490	12,980	135.9
Tarboo Lake	Jefferson	8	558	558	1116	135.8
Buck Lake	Kitsap	8	512	512	1024	136.0
Panther Lake	Kitsap	41	2775	2775	5550	135.9
Wildcat Lake	Kitsap	44	3285	3285	6570	149.1
Aldrich Lake	Mason	4	292	292	584	136.1
Benson Lake	Mason	32	2195	2195	4390	135.9
Devereaux Lake	Mason	40	2693	2693	5386	135.7
Haven Lake	Mason	28	1898	1898	3796	134.0
Robbins Lake	Mason	7	454	454	908	136.0
Tiser Lake	Mason	43	2932	2932	5864	135.9
Total			36,372	36,372	72,744	

We conducted creel surveys on 15 western Washington lowland lakes in Pierce, Kitsap, Thurston, Jefferson, and Mason counties (Table 1), ranging in area from 4.45 ha (Aldrich Lake) to 95.51 ha (Ohop Lake). These lakes support popular fisheries on the opening day of trout fishing (4th Saturday in April). Species composition varies between lakes but includes centrarchids, cyprinids, cottids and wild, native anadromous species such as coastal cutthroat trout and coho salmon *O. kisutch*.

Angler interviews were conducted from 08:00 to 12:00 h on opening day (24 April 2021) at all study lakes to estimate the catches of triploid and diploid trout. As reported by Losee and Phillips [25], this sampling period coincides with the peak of inland trout harvest in western Washington and thus the best index of the fishing season. Samplers interviewed anglers and recorded both boat and shore angler trip time, lure type, and numbers of fish caught and released, and retained. All retained fish were checked for clipped (triploid) and non-clipped (diploid) adipose fins. Informative flyers notified anglers of the presence and identification of acoustically tagged fish, and how to report and return tags that were recovered. This information was shared in a WDFW blog (https://wdfw.medium.com/the-secret-lives-of-rainbow-trout-36a2d00fd9bf) to encourage anglers to report caught trout.

### Acoustic tracking

**Fig. 1:**
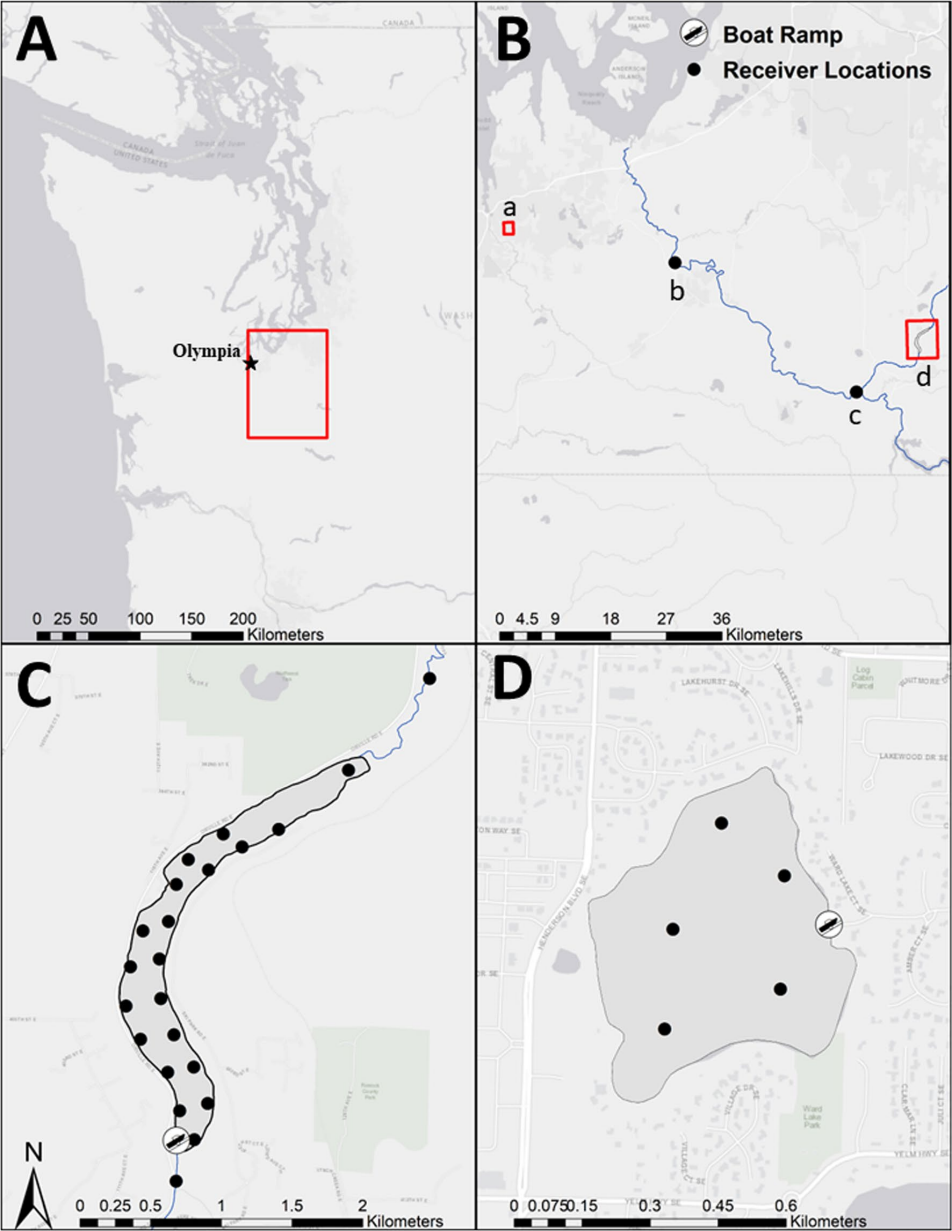
Study area map **A** indicating the area in Washington where both study lakes were located. Panel B shows Ward Lake (a) located in Thurston County, Washington, andOhop Lake (d) located in Pierce County, Washington. Also, shown in panel **B** are the two additional receivers located downstream of Ohop Lake (b, c): one receiver is located at the confluence of Ohop Creek with the mainstem as the Nisqually River (c) and a second at river km 19 of the mainstem Nisqually River (b). All other receivers in Ohop Lake are shown as black dots in panel **C**. The five Ward Lake receivers are shown as black dots in Panel **D**

The acoustic tracking component of this study took place in two of the 15 lakes, Ward (N 47.008767°, W-122.875442°) and Ohop (N 46.905224°, W-122.273341°) lakes (Fig. 1). Triploid (*n* = 40) and diploid (*n* = 40) trout were acoustically tagged (V9-6L, signal delay of 220–340 s, battery life 912 days, Innovasea, Canada, Halifax) at the hatchery. Specifically, trout were anesthetized with MS-222 (0.07 g/L) and supported upside down by a closed cell foam block during surgery, during which they were given anesthetic by gravity feed over the gills (0.02 g/L). After an incision was made in the abdomen forward of the pelvic girdle muscle, a transmitter was inserted, antibiotic injected (25 mg/kg oxytetracycline), and the incision sutured with 2–3 stitches (4-0 RB-1 Taper antibacterial Ethicon Vicryl Plus violet braided, Johnson & Johnson, United States, New Brunswick, New Jersey). The incision was treated with antibacterial ointment (Bacitracin^®^), and weight and length were recorded. Following tagging, fish were held with aerated water until swimming upright and responsive. All tags and surgery tools were disinfected with Nolvasan^®^ (chlorhexidine diacetate) and rinsed in saline solution before use and between fish. Tagged triploid fish ranged from 122 to 377 g (mean ± SD: 207 ± 45.5) and length (mm) 222–292 (mean ± SD: 250.23 ± 14.37). Diploid fish weight ranged from 128–376 g (mean ± SD, 260.0 ± 20.3) and length 227–300 mm (mean ± SD, 225.5 ± 62.3). Individuals were only tagged if they weighed more than 120 g to ensure that the internal tag did not exceed 3% of the dry body weight of the fish [35]. Prior to stocking, individuals were placed in a recovery tank and monitored for 30 d before being transported and stocked in the study lakes. Twenty triploids and twenty diploids were stocked each in Ohop Lake and Ward Lake on 20 April, on the same day as untagged individuals (Table 1), 4 days prior to opening day of fishing.

Ward Lake in Thurston County, Washington (27.11 ha, 20.4 m maximum depth) is a mixed species fishery managed for kokanee *O. nerka* and as a put-and-take fishery for rainbow trout. In Ward Lake, stocking of adult rainbow trout as a put-and-take fishery has occurred annually since 1935. Additional species found in the lake include rock bass *Ambloplites rupestris*, largemouth bass *Micropterus salmoides*, bluegill *Lepomis macrochirus* and coastal cutthroat trout (WDFW, unpublished data). Ohop Lake in Pierce County, Washington (area: 95.51 ha, maximum depth: 7.6 m) is managed as a mixed species fishery with a rainbow trout emphasis. Rainbow trout have been stocked in Ohop Lake since 1995 to provide put-and-take fishing opportunity. Additional species found in the lake include brown bullhead *Ameiurus nebulosus*, largemouth bass, largescale sucker *Catostomus macrocheilus*, sculpins *Cottus* spp., yellow perch *Perca flavescens*, pumpkinseed sunfish *Lepomis gibbosus*, black crappie *Pomoxis nigromaculatus*, coho salmon and cutthroat trout (WDFW, unpublished data). The southern end of Ohop Lake flows through Ohop Creek into the Nisqually River (Fig. 1).

Five acoustic receivers (VRTx, Innovasea, Canada, Halifax) were deployed in Ward Lake and 22 in Ohop Lake on April 19, 2021 (Fig. 1). Internal synchronization tags were used to synchronize receiver internal clocks. Prior to deployment of the receiver arrays, range testing was conducted using the same acoustic transmitters being implanted into study fish (V9-6L, signal delay of 220–340 s). In Ward Lake range testing suggested targeting 200 m to achieve a detection range greater than 90%. To achieve a detection range of 80% we targeted 150 m in Ohop Lake. Receivers were deployed approximately 230 m apart in WardLake and 200 m apart in Ward Lake. To detect fish leaving Ohop Lake we deployed one receiver at the confluence of Ohop Creek and the Nisqually River (Fig. 1) and one receiver in the lower mainstem of the Nisqually River (46.98, − 122.64). Detection probabilities for receivers varied between the lakes. In Ward Lake detection probabilities were > 70%, up to 220 m from a tag (Ward Lake; mean ± SE, 85 ± 15%) and > 50% when 130 m away in Ohop Lake (mean ± SE, 85 ± 15%).

### Data analysis

We evaluated the contribution of each ploidy strain to the catch by summing the total number of triploid (adipose fin clipped) and diploid (unclipped) rainbow trout reported to be caught during creel surveys at study lakes on opening day. A chi-square test was used to assess the probability of capture for triploids relative to diploids with the odds ratio, ɸ = Ѡ1/Ѡ2, where Ѡ1 represents the relative contribution of stocked fish from each group (triploid versus diploid) to the total stocked and Ѡ2 represents the relative contribution of fish caught in the test fishery from each group to the total number of fish caught.

Acoustic telemetry was used to detect tagged trout in Ohop and Ward lakes and estimate the rates of mortality and emigration. Angler reporting of tagged fish caught, and detection history allowed for an assignment of “fate” for individuals removed from the lake. Individuals that were last detected at the receiver in the outlet and then never detected in the lake again were classified as migrants. Tagged fish returned by anglers were classified as having been caught. Sedentary fish, based on acoustic detections, were classified as natural mortalities. All other tags that went undetected during the study period were classified as “unknown removal”.

### Fine-scale positioning

Raw acoustic telemetry detection data were downloaded and sent for processing to Innovasea for VEMCO Positioning System (VPS). VPS utilizes hyperbolic positioning to get a weighted-average position for a fish based on the time difference of arrival at multiple receivers for a single ping of a transmitter. VPS provides an estimate of the horizontal position error (HPE) associated with each of the positions [36]. Differences in space use between triploid and diploid trout were determined using fine-scale positions and kernel utilization distribution (KUD), which describes the probability of a rainbow trout in a location of the lake based on a utilization distribution [42]. Areas of high importance, known as core areas, were represented by 50% of the KUDs. Home ranges were signified by 95% KUDs. The “ks” package in R was used to calculate both home ranges and core areas for both ploidy strains and for day and nighttime periods. Day and night were defined using the “suncalc” package in R, defined by local sunrise and sunset. Individuals with fewer than 50 detections were excluded from the analysis because the data were insufficient to accurately determine a KUD. ArcGIS 10.8.2 was used to create kernel density maps for both ploidy strains and time periods (day and night) at both study lakes to qualitatively visualize the spatial distribution of the fine-scale positional data. Kernel density rasters had an output cell size of 0.1 m and show the least to most dense areas of use by each rainbow trout and between the two time periods. The home range distributions were not normally distributed, so we compared triploids and diploids in each lake and between day and night periods with a series of Mann–Whitney-Wilcoxon tests.

## Results

**Fig. 2:**
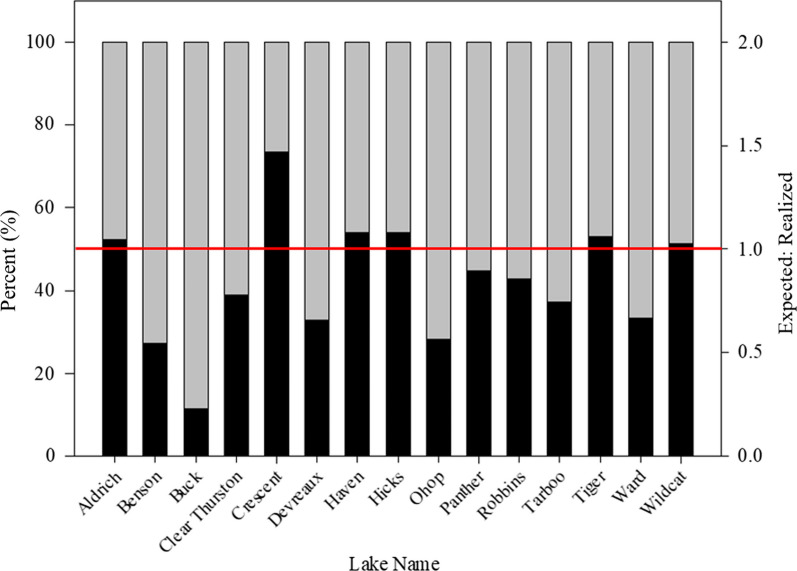
Relative proportion of triploid (black) *versus* diploid (grey) caught in selected Western Washington Lakes. Horizontal red line represents a 1:1 ratio between expected and realized for triploid catch

A total of 891 anglers were interviewed across the 15 study lakes where similar densities of triploid and diploid rainbow trout were stocked (Table 1). On opening day of fishing (April 24th) creel samplers reported 742 trout total, of which fewer were triploid (316, 42.59%) than diploid (426, 57.41%; Chi-square = 15.8, *p* < 0.001). Odds ratio revealed that across the 15 lakes stocked, triploid trout were caught at a rate 15.3% lower than would have been expected based on stocking. Lake specific patterns of trout contribution (triploids *versus* diploids) varied; diploids contributed more than triploids in 6 of 15 lakes (*p* < 0.05, Chi-square test; Fig. 2), and triploids contributed significantly more only in Crescent Lake (73.5% of observed catch, Chi-square = 15.8, *p* < 0.05; Fig. 2). In 8 of 15 study lakes, triploid and diploid trout contribution rates did not differ from expected based on stocking (*p* > 0.05, Chi-square test).

**Fig. 3:**
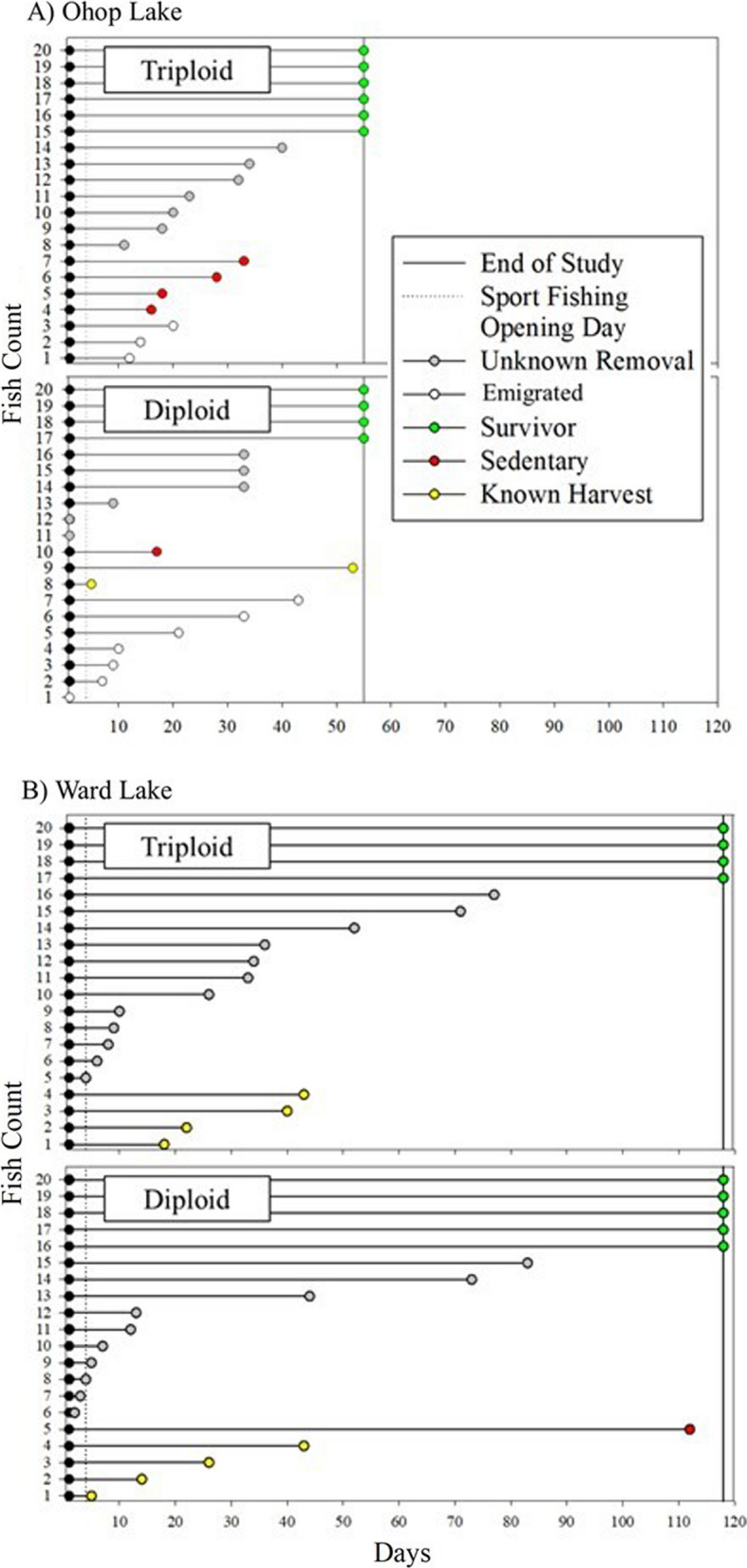
Tagged triploid and diploid rainbow trout across the study period from 24 April–23 August 2021 in **A** Ohop Lake, Pierce County and **B** Ward Lake, Thurston County, Washington. Each horizontal line represents an individual fish for the period that they remained in the study area, and fish are grouped by their fate in the study

**Fig. 4:**
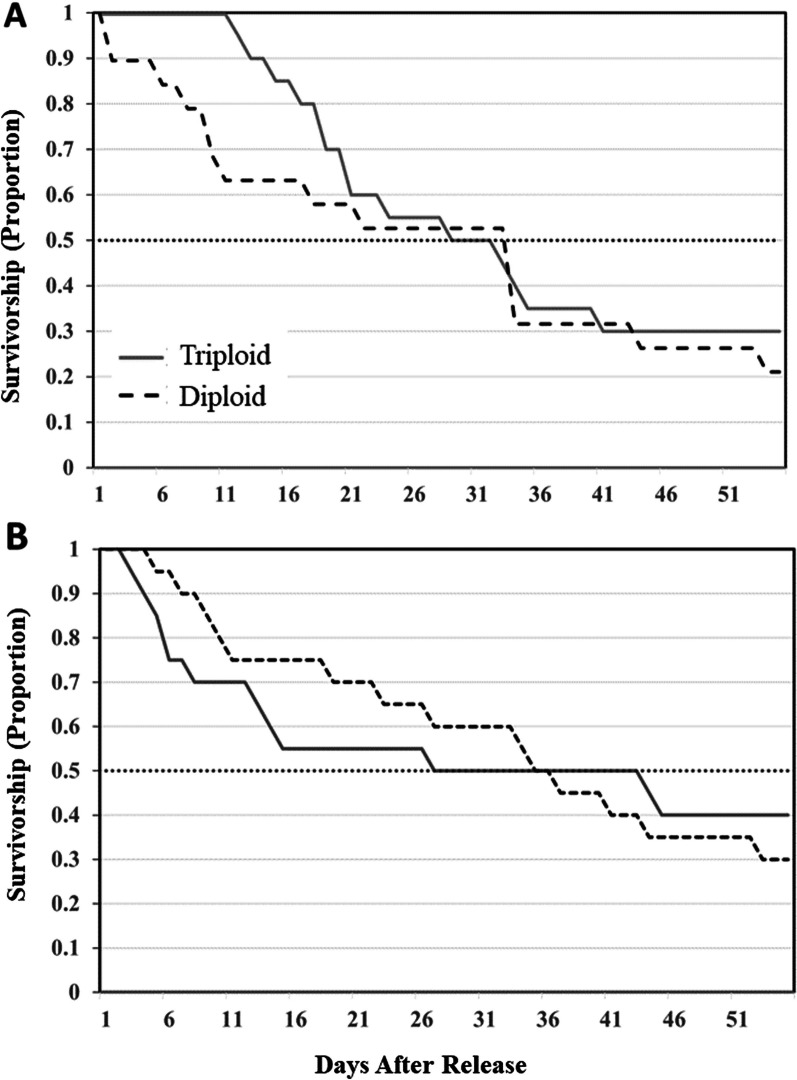
Trout survivorship for Ohop Lake (**A**) and Ward Lake (**B**) diploid (black-dashed) and triploid (grey) rainbow trout. With a dotted line indicating the time at which 50% of individuals were no longer in the study

In the two study lakes, acoustic receivers recorded more than 300,000 individual detections from the 80 tagged trout, with an average of 3,850 detections per trout (± 1802). All tagged fish were detected the first day after stocking, and 19 tagged fish were still present 55 days later, on 15 June (9 in Ward and 10 in Ohop; Fig. 3). Overall apparent survivorship was similar for triploids and diploids but different between lakes with 50% of tagged trout in Ohop Lake no longer available to the fishery 21 d after stocking because of capture, apparent natural mortality (i.e., tag became motionless in the lake), migration, or unknown removal (Fig. 4). In Ward Lake, fish survived longer; 50% were still available 36 d after stocking (Fig. 4).

Anglers reported recoveries of tagged trout in both Ward (4 diploid and 4 triploid) and Ohop Lake (2 diploid), all within two months of stocking (Fig. 3). The last detection locations indicated that 25% (10/40) of stocked trout migrated from Ohop Lake, mostly (8/10) within 21 d of stocking (Fig. 3) and mostly (7/10) diploid trout. Two diploid rainbow trout were reported as taken by anglers at Ohop Lake. Nearly half (44%: 35/80) of the tagged trout were removed from lakes by unknown causes and 24% (19/80, 10 triploids and 9 diploids) survived until the end of the study (Ward Lake: 118 days, Ohop Lake: 55 days).

**Fig. 5:**
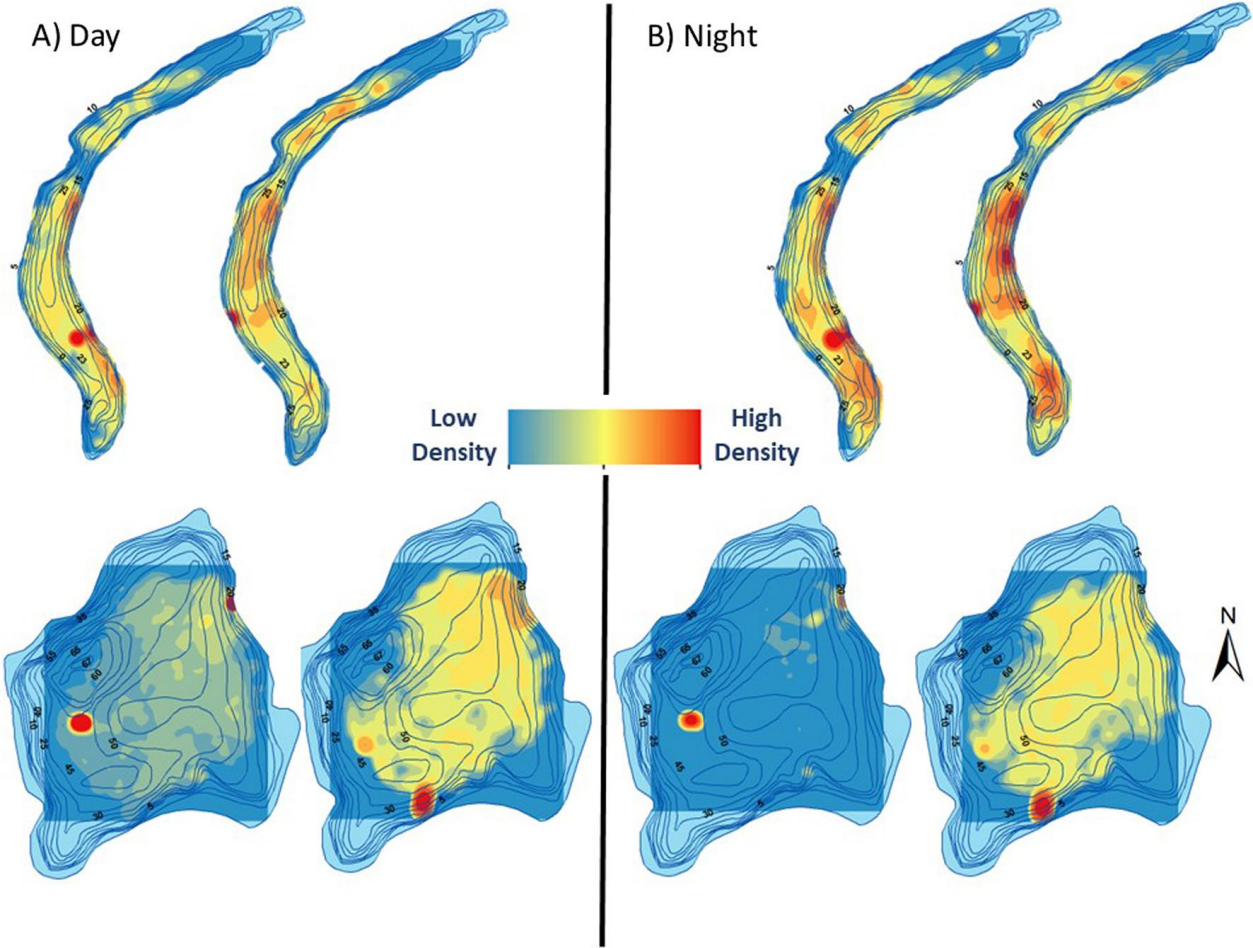
Kernel density of triploid (left) and diploid (right) rainbow trout during the day (**A**) and night (**B**) in both study lakes. Kernel density rasters had an output cell size of 0.1 m and show the least to most dense areas of use by each rainbow trout ploidy strains and between the two time periods

**Fig. 6:**
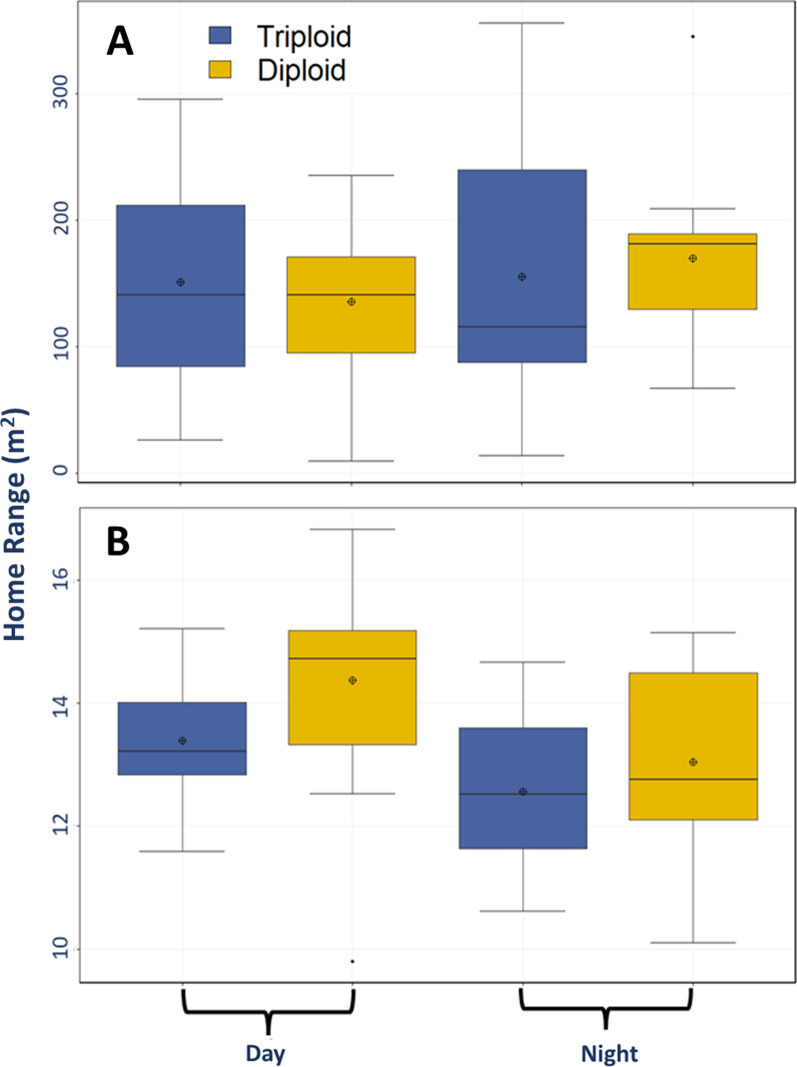
Home ranges (m^2^) for both triploid and diploid rainbow trout in both the day and night periods at Ohop Lake (**A**) and Ward Lake (**B**). Note differing Y-axis between panels. Home range did not differ significantly between diploids and triploids (Mann–Whitney–Wilcoxon test, W = 1623, *p* = 0.30) or between the two time periods (Mann–Whitney–Wilcoxon test, W = 2095, *p* = 0.15)

The HPE values for synchronization tag position data collected in the two weeks prior to the start of the study were compared to twice the distance root mean square of measured error (HPEm) [3, 28].VPS calculated positions for study fish were filtered by HPE less than 10 to significantly reduce positioning errorwhich resulted in 55.4% (58,743) of positions in Ward Lake having a HPE less than 10. In Ohop Lake 75.4% (27,264 positions) had an HPE less than 10. Qualitative spatial analysis of the data indicated more variability in lake usage areas for diploid trout in comparison to triploid trout. However, the time of day did not greatly impact the patterns of usage (Fig. 5). Areas of high use were focused on the central portions of both study lakes with fish moderately using some littoral regions of the lake (e.g., southern shore of Ward Lake and eastern shore of Ohop. Overall, home range did not differ significantly between diploids and triploids (Mann–Whitney–Wilcoxon test, W = 1623, *p* = 0.30 or between day and night periods (Mann–Whitney–Wilcoxon test, W = 2095, *p* = 0.15; Fig. 6). Home ranges were significantly greater for both diploids and triploids in Ohop Lake than Ward Lake (Mann–Whitney-Wilcoxon test, W = 3522,* p* < 0.005, Fig. 6), likely because Ohop Lake is larger (95.51 ha vs. 27.11 ha for Ward Lake). In neither lake was there a significant difference in ploidy strain (Mann–Whitney–Wilcoxon test, Ward: W = 350, *p* = 0.05; Ohop: W = 349, *p* = 0.27) or time period (Mann–Whitney–Wilcoxon test, Ward: W = 604, *p* = 0.14; Ohop: W = 426, *p* = 0.78).

## Discussion

The results of this multi-faceted study, combining standard creel monitoring with fine scale tracking, indicated that triploid trout were a viable alternative to traditional diploids for maintaining angler opportunity while reducing the conservation concern associated with genetic introgression. Standard creel monitoring in 15 lakes showed that catch rate of diploids was greater than 50% in most lakes but triploid trout still contributed greatly to fisheries. Secondly, fine scale tracking showed that triploids had a decreased rate of emigration out of the lake, similar survivorship, and similar diel movements in comparison to diploid trout. However, the small number of trout leaving reduced our ability to demonstrate a difference in migration (3 triploids vs. 7 diploids), and this might be fruitful area of future work.

Our study represents the first use of acoustic telemetry to document the movement patterns of triploid rainbow trout and provides important insights into the catchability of stocked triploid trout relative to traditional diploids. Consistent with previous studies comparing catch rates of triploid *versus* diploid trout, our results indicated that triploid trout stocked in lakes can return to the creel at a somewhat reduced or similar rate than that of diploids [9, 21, 22]. By combining standard creel monitoring and fine scale acoustic telemetry, our results help to understand why rates of catchability between triploids and diploids often differ. Specifically, fewer triploid trout left the lake, and remaining trout had similar home ranges between the two ploidy strains; both these qualities may be perceived as desirable for fisheries management objectives associated with the need to balance conservation and fishing objectives. These findings have important implications for managers weighing the cost and benefits of differing stocking plans.

In Ohop Lake, where migrating rainbow trout have access to waters used by anadromous conspecifics, 25% (10/40) of the tracked trout were last detected in the outlet of the lake. Extrapolating the observed rate of migration to the total number of trout stocked in Ohop Lake (12,980), as many as 3245 hatchery trout might have left Ohop lake in 2021. The present study had a relatively small sample of tracked fish, therefore, we recommend caution in such an extrapolation, and regard these results as tentative. None of the tracked rainbow trout were detected at the confluence of Ohop Creek and Nisqually River or in the lower Nisqually River, so it is likely that stocked trout the left Ohop Lake remained in the creek or experienced low survival in the fluvial environment of Ohop creek, consistent with other studies [2, 18, 37]. Regardless of the exact number of trout that left Ohop Lake in the current study, rates of emigration are significant in Ohop Lake. Risks associated with these findings (e.g., competition, genetic introgression) may be partially mitigated by stocking sterile triploid trout. Trout stocking plans are designed to achieve goals based on angler opportunity and satisfaction. Therefore, stocking strategies that limit emigration and reduce gene flow from domesticated hatchery stocks to wild trout while achieving these angler-related goals are preferable.

Triploid trout stocked in Ohop and Ward lakes demonstrated comparable home ranges relative to diploid trout. However, density maps (Fig. 4) showed less variability in the distribution of triploid home ranges, perhaps further limiting the potential to leave the waterbody they were stocked in, relative to diploids. This reduced migration rate for triploids may provide a benefit for fisheries managers. Additionally, the reduced variability in home range may have contributed to the slightly overall lower catch rates for triploids observed in the current study, if diploids distributed in a way that enhanced their potential to be caught. Given the conservation concern associated with wild steelhead and cutthroat trout in waterbodies connected to important put-and-take rainbow trout fisheries [39], managers may benefit from prioritizing the available triploid rainbow trout for stocking in lakes where both the conservation risks and likelihood of emigration are the greatest. In addition, consideration should be given to the potential for mitigating for reduced catch of triploids by considering other factors that influence catch rates, such as stocking density [29], stocking season [44], prey availability [13], fish size [7, 25] and stocking location [15] to fine-tune triploid stocking plans. Together these results suggest raising fish to a larger size, stocking near fishing access points and stocking just prior to the opening of the fishery are likely to support a reduction in the total fish that need to be released, thus mitigating increased cost or reduced catch rate associated with stocking triploids. In doing so, managers could maximize chances of achieving management objectives associated with both conservation and opportunity.

Our study was not designed to identify causes of variability in catch rates between triploid and diploid trout. However, others have explored this topic and the results have important management implications to consider before applying these results to other systems. Previous studies suggested that triploids may have a reduced aerobic capacity and decreased tolerance to chronic stress [12, 33], therefore catch rates and movement patterns could be affected by variability in habitat conditions (e.g. temperature, pH). In the current study, the catch rate of diploids was greater than 50% in most study lakes over a broad range of environmental conditions. For example, diploids made up > 75% of trout sampled in one of the smallest lakes in this current study, Buck Lake (7.69 ha) and the largest lake, Ohop Lake (131.93 ha) suggesting lake size alone is not a good predictor of triploid trout catchability. Koenig et al. [21] found stocking density to be the most important factor explaining variability of triploid trout catchability, but we observed differences in movement patterns and catch for tagged triploids relative to diploids across two different sized lakes stocked at similar density and variable catch rates across the broader set of lakes. This information highlights the need to better understand factors affecting catch rates of triploid rainbow trout to increase precision around stocking programs. While it is beyond of the scope of this study, future work should further investigate factors affecting both catch rate and home range of triploids and diploids to clarify potential causes for the patterns reported here.

Triploid rainbow trout represent an important tool for fisheries managers faced with increasing threats to wild populations of salmonids and growing pressure for fisheries managers to design sustainable fishing opportunity. Pairing acoustic telemetry with a traditional stock assessment tool (i.e., creel survey), we demonstrated that triploid trout were a viable alternative when stocking rainbow trout in western Washington lakes. Compared to diploid trout, triploids were caught at a reduced rate overall but exceeded or met expectations in many waterbodies (Fig. 2). With a comparable home range and reduced rate of emigration, our results provide support for a modification of trout stocking where concerns over genetic introgression with wild stocks exist [32, 39]. A strategic approach by managers to integrate triploids into current stocking plans while prioritizing values (e.g. conservation vs. opportunity) has potential for maintaining or improving catch rates of these popular sport fisheries while providing increased protection for native populations.

## Data Availability

The data that support the findings of this study will be available upon request.
